# Intestinal Strongyle Genera in Different Typology of Donkey Farms in Tuscany, Central Italy

**DOI:** 10.3390/vetsci7040195

**Published:** 2020-12-02

**Authors:** Michela Maestrini, Marcelo Beltrão Molento, Simone Mancini, Mina Martini, Francesco Giovanni Salvo Angeletti, Stefania Perrucci

**Affiliations:** 1 Department of Veterinary Sciences, University of Pisa, Viale delle Piagge 2, 56124 Pisa, Italy; simone.mancini@unipi.it (S.M.); mina.martini@unipi.it (M.M.); francesco.angeletti@phd.unipi.it (F.G.S.A.); 2 Department of Veterinary Medicine, University of Paraná, R: dos Funcionarios, 1540, Curitiba 80035-050, PR, Brazil; molento@ufpr.br

**Keywords:** intestinal strongyles, donkey, cyathostomins, *Cylicocyclus* spp., *Cylicostephanus* spp., central Italy

## Abstract

Intestinal strongyles are common helminths of donkeys, in which they may be responsible for disease and poor performance. This study aimed to identify intestinal strongyle genera in 55 naturally infected donkeys from three different farm typologies in Tuscany, central Italy, using morphological and metrical analysis of third stage larvae (L3) obtained from faecal cultures. Larvae were identified using two previous reported morphological identification keys. Moreover, eggs per gram (EPG) data were also evaluated to assess differences, if any, according to the farm typology, sex, and age of the examined donkeys. The results showed that small strongyles were prevalent in all donkey farms. In all examined farms, most (92–100%) of L3 were identified as cyathostomin species of the genera *Cylicocyclus* spp. and *Cylicostephanus* spp. Large strongyles of the genera *Strongylus* spp. and *Triodontophorus* spp., were identified at low percentage (8%), only in the single organic farm included in the study. A high agreement was observed between the two different morphometric keys used. No significant differences were found for EPG according to farm typology, and sex and age from the examined donkeys. This is the first report about genera identification of intestinal strongyles infecting donkeys in Tuscany, Italy.

## 1. Introduction

Donkey breeding has always been considered a marginal livestock production worldwide. However, in recent years in Europe, and mainly in Italy, donkey breeding has become more popular [1]. In Italy, donkeys are primarily reared to produce milk intended for human consumption. Due to its similarity to the human breast milk, donkey milk is used in neonatal nutrition for children allergic to cow’s milk [2]. Moreover, due to its high content of essential fatty acids and vitamins A, B, C, and E, endowed with an epithelial-protecting activity, donkey milk is also used in cosmetics [3]. In the Italian territory, currently there are about 35 dairy donkey farms in which about 700 dairy donkeys of different breeds (i.e., Romagnola, Amiatina) are reared [1]. In Italy, donkeys are also employed in social activities, such as onotherapy and tourism, or as in the other European countries, they are used as working animals in marginal areas [1]. In Tuscany, central Italy, many donkey farms have less than five donkeys per farm, while about 100 donkeys are raised in only seven farms, consisting of more than five animals [4,5].

As in the other equid species, endoparasites, especially helminths, may represent a threat to the welfare and production of infected donkeys [6]. Prevalent donkey helminths include the intestinal roundworm species *Parascaris* spp. and intestinal strongyles, that in infected donkeys may be the cause of weight loss and reduced growth and production, decline in general conditions, intestinal obstruction, colic episodes, and diarrhoea [6]. In previous studies, a prevalence of donkey intestinal strongyle infections ranging from 72% to 100% has been reported in Europe [6,7]. Intestinal strongyles may be distinguished in two subfamilies, namely the Strongylinae, which includes mainly species of the genera *Strongylus* and *Triodontophorus*, also known as large strongyles, and the Cyathostominae, which consists of a complex group of more than 50 species, known as small strongyles [6]. In the last years, cyathostomins have been more frequently reported in European donkey farms [6].

Adults of all intestinal strongyles live in the intestinal lumen of the large intestine of equids but, while larvae of cyathostomins and *Triodontophorus* spp. are encysted in the large intestinal wall, the larvae of *Strongylus* spp. show a migratory pattern [8]. Both, adult and larval stages of intestinal strongyles play an important role in the epidemiology and pathogenicity of these infections. These parasites may cause weight loss, colic and, rarely, diarrhoea in infected donkeys [6,9,10,11,12,13,14]. Within the genus *Strongylus*, four species that affect donkeys are recognised: *Strongylus vulgaris*, *Strongylus edentatus*, *Strongylus equinus*, and *Strongylus asini*. *S. vulgaris* is considered the most pathogenic species, as this parasite can be responsible for thromboembolic colic and, frequently, for the death of heavily infected animals [8]. In fact, in the life cycle of *S. vulgaris*, third stage larvae (L3) invade the intestinal mucosa, moult to the fourth stage, penetrate local arterioles and migrate to reach the cranial mesenteric artery and other numerous blood vessels arising from the aorta [8]. After about 4 months, *S. vulgaris* mature larvae return to the large intestine lumen where they become adult parasites [8].

In *S. edentatus* and *S. equinus* life cycles, larval migration is limited to the liver and peritoneum and to the pancreas and liver, respectively. *S. asini* is a common internal parasite species of zebras and donkeys in Africa and this species morphologically resembles *S. vulgaris*, but it is genetically more closely related to *S. edentatus* and *S. equinus* [12,13]. Adults occur in the cecum and colon, but different larval stages migrate within the liver, possibly causing hepatic cysts that are frequently reported in zebras [12]. The larvae may be found attached to the lining of hepatic and portal veins [13].

Although molecular identification tools are available [12,15,16], adults of all intestinal strongyle species can be differentiated by morphological characters [17,18,19,20,21]. Eggs passed in faeces are, however, indistinguishable unless cultured to produce L3 that can be morphologically identified at the genus or species level [19,21]. Data about intestinal strongyles infecting donkeys in Italy and elsewhere are limited, and only few studies reported the identification of cyathostomins in animals infected by intestinal strongyles [22,23,24]. This study aimed to determine the morphological identification of L3 of intestinal strongyles infecting donkeys in different typology farms in Tuscany, Italy.

## 2. Materials and Methods

### 2.1. Animals and Farms

Fifty-five donkeys of the Italian breeds, Romagnola, Amiatina, and cross-bred animals of both sexes and different age (from 10 months to 15 years old), reared outdoors in a semi-extensive system with free access to pastures, were used in this study. The examined animals were from three different typology farms, located in Tuscany, central Italy. More precisely, these farms were from the provinces of Arezzo, Pisa, and Grosseto. In the farms in Arezzo and Grosseto, donkeys were reared mainly for production of pasteurised milk intended for human consumption. In the Arezzo farm, donkeys were used also for recreational and social activities. This latter farm was also the only organic donkey farm examined in this study. In Pisa, donkeys were used mainly for onotherapy and other social and recreational activities.

No treatments were performed in the dairy organic farm included in the study, because of the limitation given by the European legislation. Selective treatment with 50 mg/kg of fenbendazole *per os* (Panacur pasta cavalli Sir 24 g, Intervet Italia S.r.l., Segrate, Milan, Italy), was given to animals of the dairy donkey farm in Grosseto, based on results of faecal egg count (FEC) analysis. In the farm in Pisa, all animals were treated once a year in spring with an oral commercial formulation containing ivermectin and praziquantel (Equimax Gel Os Sir 7,49 g, Virbac Srl, Milan, Italy).

According to the farm of origin, examined donkeys included 16 lactating jennies from the Grosseto farm, 29 donkeys (14 jennies, 1 jack, 1 gelding, 9 fillies, and 4 foals) reared in the Arezzo farm and 10 adult donkeys of both sexes (6 jennies and 4 geldings) reared in the farm of the province of Pisa. All donkeys were submitted to a rapid clinical examination by a registered veterinarian and the state of nutrition and the body condition score (BCS) of the animals was assessed [25].

### 2.2. Parasitological Analysis

Faecal samples were collected directly from the rectal ampulla of each donkey. Animals from Grosseto and Pisa had been treated 6 months prior the sampling. Samples were examined by the simple flotation test and the McMaster technique with a sensitivity of 50 eggs per gram (EPG) of faeces, using saturated NaCl as a flotation solution (specific gravity of 1.2) [23,26]. To obtain the L3 of intestinal strongyles, faecal cultures were prepared with single or pooled faecal samples with >200 EPG, in order to determine more larval variability for all parasite species, especially considering the current low occurrence reported for *S. vulgaris* (<2%) in previous reports [27]. The faecal pools were made using samples from two or three different animals of the same farm, sex, age, and productive status. In some cases, more than one pool was made with the same faecal samples. At least 10–15 faecal cultures were examined for each farm. For the faecal cultures, about 40 g of faeces were placed on a gauze stretched inside a plastic cup containing about 20 mL of water and covered with the bottom of a perforated plastic cup to allow proper oxygenation. Faecal cultures were incubated at 26.5 °C for 10–14 days to allow complete larval development. During the incubation period, cultures were sprayed with distilled water to ensure adequate humidity conditions. After the incubation time, L3 were collected using the Baermann technique [26].

Harvested L3 were examined in two different ways, fresh or stained with 2% Lugol iodine solution, covered with a coverslip, and observed under optical microscopy at different magnifications (from 40 to 400×). L3 from each culture were microscopically examined and identified using two different previous reported morphological identification keys, based on the total body length and width, intestinal and oesophageal length, and on the number, arrangement, and shape of intestinal cells (IC) [19,21].

### 2.3. Statistical Analysis

Quantitative data obtained from faecal analysis taken from all donkeys reared in the four farms were statistical examined by one-way ANOVA, in order to assess possible differences about the EPG according to age/sex classes of jennies, jacks and geldings, fillies and foals. Moreover, quantitative data (EPG) observed in jennies of the organic and the conventional dairy farms were also compared. Prior to statistical analysis, EPG data were log 10 transformed to meet normality distribution. Statistical significance was set at *p* < 0.05.

### 2.4. Ethical Statement

The study was carried out following the recommendations of the European Council Directive 155 (86/609/EEC) on the protection of animals and in adherence to a high standard of veterinary care. Ethical approval was not required in this study, as faecal sampling was performed by the veterinarians of the farms, as part of their routine clinical visit and parasitological monitoring protocols.

## 3. Results

**Figure 1:**
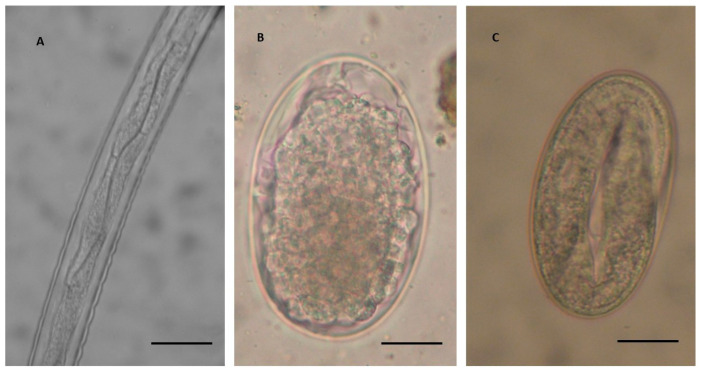
Detail of the body of third stage larvae (L3) of cyathostomin (**A**), showing eight intestinal cells of which the first four are arranged in a double row and the second group of four cells are in a single line (magnification 400×). This typology of L3 was identified as *Cylicostephanus* spp. (*Cylicostephanus hybridus, Cylicostephanus calicatus and Cylicostephanus longibursatus*) according to Bevilaqua et al. [19], and as type C L3 according to Santos et al. [21]. Scale bar: 50 µm. (**B**) Undeveloped cyathostomin egg and (**C**) developed cyathostomin egg containing a first stage larva (magnification 400×). Scale bars: 20 µm.

All examined donkeys were apparently asymptomatic, and BCS and other examined parameters were in normal range (data not shown). All donkey faecal samples (100%) from all examined farms were found positive for intestinal strongyle eggs (Figure 1B,C). More specifically, animals had 450 EPG of intestinal strongyles in the farm in Grosseto (from 150 and 1900), 500 EPG in the farm located in Arezzo (from 50 and 2400), and 300 EPG in the farm located in Pisa (from 50 and 750). However, no statistical differences were found according to farm typology, age, and sex of all examined animals (*p* > 0.05). Additionally, no statistical differences emerged by the comparison of the EPG counts observed in jennies of the two dairy farms included in this study (*p* > 0.05).

In all farms (Table 1), L3 obtained from faecal cultures revealed a high percentage of cyathostomins showing an elongated body, a tapering anterior portion, a long tail ending with a long filament, and IC clearly distinguishable under the microscope. Table 1 summarises the percentages of the different genera of small and large strongyles according to the morphological keys given by Bevilaqua et al. [19].

**Figure 2:**
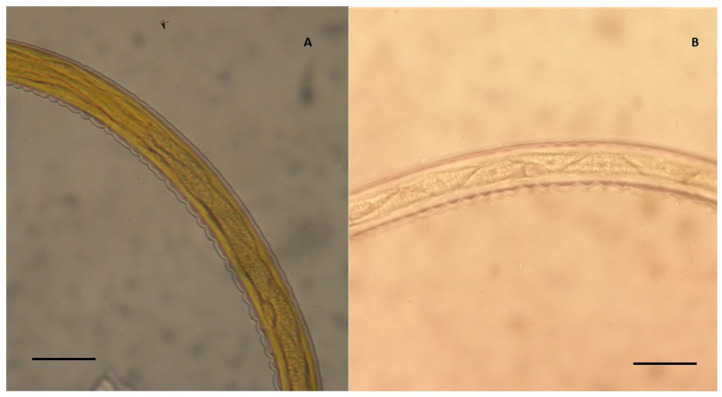
Detail of the body of two cyathostomin third stage larvae (L3). (**A**) L3 showing eight triangular intestinal cells, where the first two are arranged in a double row and the remaining six cells in a single row (magnification 400×), identified as a group 2 L3 (*Cylicocyclus nassatus*, *C. radiatus*, *C. insigne*, *Cylicostephanus minutus,* and *Cyl. poculatus*) according to Bevilaqua et al. [19], and as a type A L3 (*C. nassatus*, *C. insigne*, *C. radiatus*, *Cyl. minutus*, *Cya. catinatum*, *Cya. pateratum*, *Petrovinema poculatum*) according to Santos et al. [21]. (**B**) Type G L3 according to Santos et al. [21], showing eight trapezoidal intestinal cells with an uncharacterised arrangement. Scale bars: 50 µm.

According to the keys reported by Bevilaqua et al. [19], a first group of species belonging to the genus *Cylicostephanus* (*C. hybridus, C. calicatus,* and *C. longibursatus*) were identified. These larvae are characterised by eight IC, of which the first four arranged in double row and the remaining six IC arranged in a single row (Figure 1). The second group of cyathostomins were from *Cylicocyclus* and *Cylicostephanus* (*Cy. nassatus*, *Cy. radiatus*, *Cy. insigne*, *C. minutus*, and *C. poculatus*), and were characterised by eight IC, of which the first two arranged in double row and the remaining six in a single row (Figure 2A).

All L3 were identified in the farms of Grosseto and Arezzo (Table 1). More precisely, the percentages of the first and the second group of L3 were about 25% and 75% in the farm of Grosseto and 24% and 68% in the farm of Arezzo, respectively. However, in the farm of Arezzo approximately 8% of L3 were identified as *Strongylus* (5%) and *Triodontophorus* (3%) (Table 1).

In the farm from Pisa (Table 1), morphological analysis of L3 obtained from faecal cultures revealed that 60% of L3 belonged to the first group of larvae found in the previous two farms, while about 34% to species of the second group (Table 1). However, in this farm about 2% of L3 were identified with a third group composed by larvae showing eight IC arranged in a double row, including *Cy. ultrajectinus*, *Cy. brevicapsulatus,* and *C. bicoronatus*, while the remaining 4% were identified as *Strongyloides westeri*.

According to the keys reported by Santos et al. [21], type A and type C L3, including *Cy. nassatus*, *Cy. insigne*, *Cy. radiatus*, *C. minutus*, *Cya. catinatum*, *Cya. pateratum*, *Petrovinema poculatum*, *C. hybridus*, *C. calicatus*, were identified in all examined farms. A small number of type B (*Cy. ultrajectinus*, *Cy. brevicapsulatus,* and *C. bicoronatus*) and type G L3 (Figure 2B), were identified only in the farm in the province of Pisa.

## 4. Discussion

It was evidenced in this study, a higher prevalence of cyathostomins as generally observed in other European donkey farms [6,7,22,23]. In the present study, no differences in EPG counts were observed according to the age of examined donkeys. In equids, intestinal strongyles can be responsible for colic, diarrhoea, and in more severe cases for intestinal infarction and death, especially in horses [6,9,10,11,28,29,30]. Previous studies measuring the EPG of intestinal strongyles in donkeys was found generally higher than in horses [6,14,31], although infections tend to be higher in young donkeys [6].

Even though all the animals from the different typology of farms included in this study were found positive for intestinal strongyles, they did not show any clinical signs of parasite infection. These results would agree with some previous data showing that most of infected donkeys appear to remain clinically healthy even when a high level of infection occurs [6,14,32]. Compared to horses, little is known about the pathogenic effects of large and small intestinal strongyle infections on the health and performance of infected donkeys. However, more recently cyathostomin larvae encysted in the intestine were found to be a major cause of acute and chronic diseases and of larval cyathostominosis also in donkeys and mules [31,32,33,34]. Indeed, cyathostomins may have a negative impact on donkeys, as they can be the cause of reduced body condition and anaemia in untreated compared to treated animals, as observed by Matthee et al. [35].

Nevertheless, differences in the pathogenic effects of cyathostomin infections observed in donkeys in previous studies refer to the dependence of the different virulence of many cyathostomin species. Therefore, the correct evaluation of the prevalence and pathogenicity of cyathostomin species present in donkey farms, coupled to the identification of those species more frequently involved in clinical forms and reduced productive performances (i.e., milk production in Italian farms) in infected donkeys, may give a great help in clarifying this important topic. All these factors still have to be determined in field studies in donkeys.

Different pattern of genera composition and proportions of large and small strongyle infections have been reported in donkeys in different geographical areas. In Africa, a high prevalence of both large and small strongyles was generally found in donkeys infected by intestinal strongyles [36,37,38]. In recent studies in donkeys in Egypt and Sudan [36,38] a high prevalence of both small strongyles (cyathostomins, 72.3 to 90%) and large strongyles (84%), including *S. vulgaris* (16.7–60%), *S. equinus* (22–30%), and *S. edentatus* (18–30%), were observed. Similarly, in a large study with 2935 donkeys in Ethiopia [37], the prevalence of large strongyles was very high (*S. vulgaris* 91.3%, *S. edentatus* 0.97%, *S. equinus* 9.7%, *Triodontophorus* spp. 12.6%), although similar to that of cyathostomins (99%).

On the contrary, a higher prevalence of cyathostomin infections were found in European donkey farms [6,14]. More specifically, *Strongylus* spp. prevalence has drastically decreased over time in horses and donkeys [39], whereas cyathostomin infections have become a major issue [39,40]. Cyathostomins were also prevalent in 100% of the farms in horses in South America [41]. This widespread prevalence of cyathostomins in domestic equids is considered a consequence of the repeated use of anthelmintics (i.e., ivermectin) in the last few decades, which have applied selective pressure on large strongyles in favour of small strongyles [16,17,41,42].

The few previous data available in Italy seem to be in line with the results obtained in other European countries, since a higher prevalence of small strongyles in respect to that of large strongyles was reported from southern and northern Italy [22,24,43], while in a donkey farm in central Italy a high prevalence of cyathostomins (>90%) was evidenced [23].

Data from this study confirm these previous observations, since cyathostomins were found prevalent in all the three different typologies. More specifically, in all farms examined in this study, most of identified L3 were found to be from the cyathostomin species, mainly from the genera *Cylicocyclus* and *Cylicostephanus*. On the contrary, large strongyle species belonging to the genera *Strongylus* spp. and *Triodontophorus* spp., were identified at low percentage, but only in one out of the four farms included in the study. It is known that *S. vulgaris* is found in horses with low EPG [27]. However, in this study we did not find any correlation between low EPG counts and the higher presence of *S. vulgaris* eggs.

Nevertheless, the identification of large strongyle species confirm previous reports about large strongyles infecting donkeys in Italy [22]. Likewise, a high degree of agreement was observed between the two different morphometric keys used in this study for the identification of L3 at the genus/species level [19,21]. Interestingly, large strongyles were identified only in the organic farm included in this study, in which anthelmintic treatments are strictly limited by the European law [44,45,46]. As previously evidenced in horses [39,42], this finding reinforces that the drug restriction could be linked with the lower anthelmintic selection pressure in this latter farm in relation to the other examined farms. However, further studies are needed to confirm the effect of this type of management on the genera/species composition of donkey intestinal strongyles.

In conclusion, the results from this study are the first report regarding the prevalence of intestinal strongyle genera infecting donkeys in central Italy using two morphological identification keys. The present data represents the first step towards the potential identification of possible associations between cyathostomin species, and the occurrence of disease or the reduced performances (i.e., lower milk production) in infected donkeys.

## Figures and Tables

**Table 1:** Percentage (%) of small and large intestinal strongyle third stage larvae (L3) found in three different typologies of donkey farms in Tuscany, central Italy, identified according to Bevilaqua et al. [20].

Province/Group of L3	Grosseto ^a^	Arezzo ^b^	Pisa ^c^
Group 1 *C. hybridus*, *C. calicatus*, and *C. longibursatus*	25	24	34
Group 2 *Cy. nassatus*, *Cy. radiatus*, *Cy. insigne*, *C. minutus*, and *C. poculatus*	75	68	60
Group 3 *Cy. ultrajectinus*, *Cy. brevicapsulatus*, and *C. bicoronatus*	-	-	2

^a^ = donkeys reared for milk production intended for human consumption; ^b^ = donkeys reared mainly for milk production intended for human consumption, but also for recreational and social activities; ^c^ = donkeys used for onotherapy, recreational and social activities.
